# Editorial: Photocatalysis for Environmental Applications

**DOI:** 10.3389/fchem.2019.00303

**Published:** 2019-05-01

**Authors:** Fan Dong, Yuxin Zhang, Sen Zhang

**Affiliations:** ^1^Chongqing Key Laboratory of Catalysis and New Environmental Materials, Engineering Research Center for Waste Oil Recovery Technology and Equipment of Ministry of Education, College of Environment and Resources, Chongqing Technology and Business University, Chongqing, China; ^2^Research Center for Environmental Science & Technology, Institute of Fundamental and Frontier Sciences, University of Electronic Science and Technology of China, Chengdu, China; ^3^College of Materials Science and Engineering, Chongqing University, Chongqing, China; ^4^Department of Chemistry, University of Virginia, Charlottesville, VA, United States

**Keywords:** photocatalysis, environmental catalysis, air pollution, reaction mechanism, nanomaterials

Environmental pollution is one of the major challenges because of the rapid development of urbanization and industrialization. Considering this environmental challenge, providing a clean environment for human beings is very important for the sustainability. The nanostructured photocatalysts with intriguing physiochemical property have offered opportunities to solve the issue of environmental sustainability (Chen et al., 2019; Huo et al., 2019; Li J. et al., 2019). In recent years, significant advances have been witnessed on the synthesis and application of photocatalyst in environmental remediation (He et al., 2018a; Li et al., 2018c; Li X. et al., 2018, 2019; Wang et al., 2018c). These new photocatalysts have enabled wide applications in the air purification, wastewater treatment, and so on (Cui et al., 2018; He et al., 2018b; Li et al., 2018b; Xiong et al., 2018) The rapid development in catalysis science, nanoscience, and materials enable the significant advances in new strategies for the controlled preparation, photocatalysis reaction mechanism, and structure-activity relationship of photocatalysts (Dong et al., 2018a; Li et al., 2018a; Wang et al., 2018a,b). The structural features of photocatalysts can be further tuned to achieve enhanced photocatalytic performance in environmental applications (Dong et al., 2018b; Li X. et al., 2018; Wang et al., 2018d).

The rapid development in photocatalysis for environment has inspired this interesting Research Topic. We have invited scientists worldwide to contribute original research and review articles which could enhance our understanding of the key problems in environmental applications of nanostructured photocatalysts. The original articles describing the photocatalysts for environmental control, and for sustainable development have been accepted for publication after peered review. In this topic issue, the readers will find very interesting results covering the following aspects (1) design and synthesis of photocatalysts with new morphology and active catalytic sites; (2) photocatalysts for green synthesis; (3) photocatalysts for CO_2_ conversion to solar fules; (4) photocatalysts for wastewater treatment and air purification; and (5) revealing the photocatalysis reaction mechanism as applied in environmental problems.

For the g-C_3_N_4_ based photocatalysts, Guan et al. synthesized Ti_4_O_7_/g-C_3_N_4_ composites by a low temperature method. The enhanced photocatalytic activity for Ti_4_O_7_/g-C_3_N_4_ could be ascribed to the promoted charge separation and photoabsorption efficiency. Yang et al. fabricated a monolithic g-C_3_N_4_/melamine sponge by a cost-effective ultrasonic-coating method. The monolithic g-C_3_N_4_/melamine demonstrated high photocatalytic activity for NO removal and CO_2_ reduction. Guan et al. prepared the Ti_4_O_7_/g-C_3_N_4_ photocatalysts by a hydrolysis method. The Ti_4_O_7_/g-C_3_N_4_ exhibited remarkably improved photocatalytic activity for hypophosphite oxidation, which can be ascribed to the heterojunction structure of Ti_4_O_7_/g-C_3_N_4_ that enhanced charge carrier efficiency (Guan et al.).

Xu et al. prepared BiVO_4_ by a facile method and conducted a trapping experiment to study the free radical transformation mechanisms. They identified •OH and h^+^ as the main active radicals for oxidation. Han et al. developed a new photoelectrochemical (PEC) technology for simultaneous SO_2_ removal and H_2_ production. The enhanced H_2_ production and SO_2_ removal efficiency can be attributed to the improved charge carrier transfer after Mo doping (Han et al.). Regmi et al. reviewed recent advances on the microbial decontamination by photocatalysts and their possible mechanisms are highlighted.

Cui et al. fabricated the Ag_3_PO_4_/MoS_2_ nanocomposites and revealed that the improved performance of Ag_3_PO_4_/MoS_2_ can be ascribed to wide spectra response, efficient charge separation and enhanced oxidation capacity. He et al. developed a two-step ZnO-modified strategy to immobilize the catalyst on rGO sheets. The high ammonia degradation efficiency of ZnO/Cu/rGO can be attributed to the enhanced ROSs production efficiency and the activated interfacial catalytic sites. Shi et al. prepare high energy faceted TiO_2_ nanosheets by calcination of TiOF_2_ cubes. The 500°C-calcined sample exhibits the highest photocatalytic activity for removal of acetone owing to the high energy TiO_2_-NSs and the surface adsorbed fluorine.

Kim et al. synthesized the nitrogen doped TiO_2_ by a novel plasma electrolysis method. The 0.4 at.% N doped TiO_2_ catalyst showed the highest photocatalytic performance. Xu et al. developed a BiOCl/NaNbO_3_ p-n heterojunction by an *in-situ* method. The BiOCl/NaNbO_3_ composites exhibited much enhanced photocatalytic activity attributed to the formation of p-n junction between NaNbO_3_ and BiOCl that facilitated the charge separation (Xu et al.). Ren et al. synthesized the AgBr@Ag modified titanium phosphate composites. The AgBr@Ag/titanium phosphate exhibited higher photocatalytic activity and the photocatalytic degradation mechanisms were proposed.

She et al. reported selective activation of saturated C–H bond to generate the high-value-added chemicals on Ni-doped CdS nanoparticles. The high photocatalytic performance can be attributed to the cubic and hexagonal phases, Ni-doping and the charge carriers separation. Li et al. synthesized Au/BiFeO_3_ homojunctions via a simple method. The Au1.2-BFO showed efficient photocatalytic activity due to the hierarchical structure, SPR effect of Au particles, and the defects (Li et al.). Zhang and Liang fabricated the new 2D g-C_3_N_4_@BiOCl/Bi_12_O_17_Cl_2_ by a facile approach, which showed enhanced visible light absorption and electron-hole separation efficiency and thus highly enhanced photocatalytic activity for NO removal.

At last, as the Guest Editors of this topic issue, we would like to appreciate all the authors for the contributed articles and thank for all the referees for their comments on the manuscripts. We hope that the readers will find the results in articles of this topic issue interesting and useful for their research. Finally, we appreciate the editorial staff of Frontiers in Chemistry for their work in publishing of this topic issue.

## Author Contributions

All authors listed have made a substantial, direct and intellectual contribution to the work, and approved it for publication.

### Conflict of Interest Statement

The authors declare that the research was conducted in the absence of any commercial or financial relationships that could be construed as a potential conflict of interest.
